# Supplementary material for: Machine Learning Accelerates Crystallization for Structure Determination
Source: Angew Chem Int Ed Engl. 2026 May 4;65(25):e1218503. doi: 10.1002/anie.1218503 (PMC13266958; doi:10.1002/anie.1218503)

checkCIF/PLATON report

Structure factors have been supplied for datablock(s) lcz033

THIS REPORT IS FOR GUIDANCE ONLY. IF USED AS PART OF A REVIEW PROCEDURE FOR PUBLICATION, IT SHOULD NOT REPLACE THE EXPERTISE OF AN EXPERIENCED CRYSTALLOGRAPHIC REFEREE.

No syntax errors found. CIF dictionary Interpreting this report

Datablock: lcz033

Bond precision:	C-C = 0.0172 A		Wavelength=1.54184
Cell:	a=11.1399(2)	b=14.5492(3)	c=42.2954(8)
	alpha=90	beta=90	gamma=90
Temperature:	100 K		

	Calculated	Reported
Volume	6855.1(2)	6855.1(2)
Space group	P b c a	P b c a
Hall group	-P 2ac 2ab	-P 2ac 2ab
Moiety formula	C27 H17 Ag3 F18 N6 O2	C15 H3 Ag3 F18 N6, C12 H14 O2
Sum formula	C27 H17 Ag3 F18 N6 O2	C27 H17 Ag3 F18 N6 O2
Mr	1123.08	1123.07
Dx,g cm-3	2.176	2.176
Z	8	8
Mu (mm-1)	14.897	14.897
F000	4320.0	4320.0
F000'	4340.72	
h,k,lmax	13,17,50	13,17,50
Nref	6059	6004
Tmin,Tmax	0.138,0.225	0.035,1.000
Tmin'	0.061	

Correction method= # Reported T Limits: Tmin=0.035 Tmax=1.000
AbsCorr = MULTI-SCAN

Data completeness= 0.991 Theta(max)= 66.597

R(reflections)= 0.1166(5335) wR2(reflections)= 0.3509(6004)

S = 1.646 Npar= 506

The following ALERTS were generated. Each ALERT has the format
test-name_ALERT_alert-type_alert-level.
Click on the hyperlinks for more details of the test.

Alert level A

PLAT971_ALERT_2_A Check Calcd Resid. Dens. 1.09Ang From Ag02 6.38 eA-3

Author Response: This Alert is due to presence of residual density in the presence of heavy metal atom (Ag).

PLAT971_ALERT_2_A Check Calcd Resid. Dens. 0.83Ang From Ag02 5.70 eA-3

Author Response: This Alert is due to presence of residual density in the presence of heavy metal atom (Ag).

PLAT971_ALERT_2_A Check Calcd Resid. Dens. 0.82Ang From Ag03 5.64 eA-3

Author Response: This Alert is due to presence of residual density in the presence of heavy metal atom (Ag).

PLAT971_ALERT_2_A Check Calcd Resid. Dens. 1.06Ang From Ag03 5.43 eA-3

Author Response: This Alert is due to presence of residual density in the presence of heavy metal atom (Ag).

PLAT971_ALERT_2_A Check Calcd Resid. Dens. 1.02Ang From N00K 5.25 eA-3

Author Response: This Alert is due to presence of residual density in the presence of heavy metal atom (Ag).

PLAT971_ALERT_2_A Check Calcd Resid. Dens. 1.16Ang From N00J 5.02 eA-3

Author Response: This Alert is due to presence of residual density in the presence of heavy metal atom (Ag).

PLAT973_ALERT_2_A Check Calcd Positive Resid. Density on   Ag03   3.23 eA-3

Author Response: This Alert is due to presence of residual density in the presence of heavy metal atom (Ag).

Alert level B

PLAT097_ALERT_2_B Large Reported Max. (Positive) Residual Density   6.47 eA-3

Author Response: This Alert is due to presence of residual density in the presence of heavy metal atom (Ag).

PLAT971_ALERT_2_B Check Calcd Resid. Dens. 0.95Ang From Ag02   2.69 eA-3

Author Response: This Alert is due to presence of residual density in the presence of heavy metal atom (Ag).

PLAT971_ALERT_2_B Check Calcd Resid. Dens. 0.87Ang From Ag03   2.68 eA-3

Author Response: This Alert is due to presence of residual density in the presence of heavy metal atom (Ag).

PLAT971_ALERT_2_B Check Calcd Resid. Dens. 0.93Ang From Ag03   2.55 eA-3

Author Response: This Alert is due to presence of residual density in the presence of heavy metal atom (Ag).

PLAT972_ALERT_2_B Check Calcd Resid. Dens. 1.50Ang From F00P   -3.50 eA-3

Author Response: This Alert is due to presence of residual density in the presence of heavy metal atom (Ag) and disordered structure.

PLAT972_ALERT_2_B Check Calcd Resid. Dens. 0.97Ang From Ag03   -3.39 eA-3

Author Response: This Alert is due to presence of residual density in the presence of heavy metal atom (Ag) and disordered structure.

PLAT972_ALERT_2_B Check Calcd Resid. Dens. 1.07Ang From Ag03   -3.14 eA-3

Author Response: This Alert is due to presence of residual density in the presence of heavy metal atom (Ag) and disordered structure.

PLAT972_ALERT_2_B Check Calcd Resid. Dens. 1.49Ang From O00E -3.10 eA-3

Author Response: This Alert is due to presence of residual density in the presence of heavy metal atom (Ag) and disordered structure.

PLAT972_ALERT_2_B Check Calcd Resid. Dens. 1.15Ang From Ag01 -3.04 eA-3

Author Response: This Alert is due to presence of residual density in the presence of heavy metal atom (Ag) and disordered structure.

PLAT972_ALERT_2_B Check Calcd Resid. Dens. 1.59Ang From N00L -3.00 eA-3

Author Response: This Alert is due to presence of residual density in the presence of heavy metal atom (Ag) and disordered structure.

PLAT972_ALERT_2_B Check Calcd Resid. Dens. 0.54Ang From Ag03 -2.98 eA-3

Author Response: This Alert is due to presence of residual density in the presence of heavy metal atom (Ag) and disordered structure.

PLAT972_ALERT_2_B Check Calcd Resid. Dens. 1.63Ang From F007 -2.95 eA-3

Author Response: This Alert is due to presence of residual density in the presence of heavy metal atom (Ag) and disordered structure.

PLAT972_ALERT_2_B Check Calcd Resid. Dens. 0.64Ang From N00K -2.94 eA-3

Author Response: This Alert is due to presence of residual density in the presence of heavy metal atom (Ag) and disordered structure.

PLAT972_ALERT_2_B Check Calcd Resid. Dens. 1.03Ang From Ag02 -2.91 eA-3

Author Response: This Alert is due to presence of residual density in the presence of heavy metal atom (Ag) and disordered structure.

PLAT972_ALERT_2_B Check Calcd Resid. Dens. 0.94Ang From Ag02 -2.89 eA-3

Author Response: This Alert is due to presence of residual density in the presence of heavy metal atom (Ag) and disordered structure.

PLAT972_ALERT_2_B Check Calcd Resid. Dens. 1.20Ang From Ag02 -2.83 eA-3

Author Response: This Alert is due to presence of residual density in the presence of heavy metal atom (Ag) and disordered structure.

PLAT972_ALERT_2_B Check Calcd Resid. Dens. 0.51Ang From Ag02 -2.82 eA-3

Author Response: This Alert is due to presence of residual density in the presence of heavy metal atom (Ag) and disordered structure.

PLAT972_ALERT_2_B Check Calcd Resid. Dens. 1.09Ang From Ag03 -2.78 eA-3

Author Response: This Alert is due to presence of residual density in the presence of heavy metal atom (Ag) and disordered structure.

PLAT972_ALERT_2_B Check Calcd Resid. Dens. 0.66Ang From N00J -2.68 eA-3

Author Response: This Alert is due to presence of residual density in the presence of heavy metal atom (Ag) and disordered structure.

PLAT972_ALERT_2_B Check Calcd Resid. Dens. 0.88Ang From Ag01 -2.67 eA-3

Author Response: This Alert is due to presence of residual density in the presence of heavy metal atom (Ag) and disordered structure.

PLAT972_ALERT_2_B Check Calcd Resid. Dens. 1.11Ang From Ag01 -2.66 eA-3

Author Response: This Alert is due to presence of residual density in the presence of heavy metal atom (Ag) and disordered structure.

PLAT972_ALERT_2_B Check Calcd Resid. Dens. 1.08Ang From N00O -2.62 eA-3

Author Response: This Alert is due to presence of residual density in the presence of heavy metal atom (Ag) and disordered structure.

PLAT972_ALERT_2_B Check Calcd Resid. Dens. 1.04Ang From Ag03 -2.60 eA-3

Author Response: This Alert is due to presence of residual density in the presence of heavy metal atom (Ag) and disordered structure.

PLAT972_ALERT_2_B Check Calcd Resid. Dens. 1.02Ang From Ag03 -2.55 eA-3

Author Response: This Alert is due to presence of residual density in the presence of heavy metal atom (Ag) and disordered structure.

```
PLAT972_ALERT_2_B Check Calcd Resid. Dens.  0.89Ang From Ag02          -2.55 eA-3
```

Author Response: This Alert is due to presence of residual density in the presence of heavy metal atom (Ag) and disordered structure.

```
PLAT972_ALERT_2_B Check Calcd Resid. Dens.  1.23Ang From Ag02          -2.55 eA-3
```

Author Response: This Alert is due to presence of residual density in the presence of heavy metal atom (Ag) and disordered structure.

Alert level C

```
DIFMX02_ALERT_1_C  The maximum difference density is > 0.1*ZMAX*0.75
          The relevant atom site should be identified.
PLAT042_ALERT_1_C Calc. and Reported MoietyFormula Strings  Differ     Please Check
              Calc: C27 H17 Ag3 F18 N6 O2
              Rep.: C15 H3 Ag3 F18 N6, C12 H14 O2
PLAT082_ALERT_2_C High R1 Value ..................................       0.12 Report
PLAT084_ALERT_3_C High wR2 Value (i.e. > 0.25) ...................       0.35 Report
PLAT234_ALERT_4_C Large Hirshfeld Difference F00P     --C01C     .       0.16 Ang.
PLAT342_ALERT_3_C Low Bond Precision on  C-C Bonds ...............    0.01725 Ang.
PLAT906_ALERT_3_C Large K Value in the Analysis of Variance ......      4.403 Check
PLAT911_ALERT_3_C Missing FCF Refl Between Thmin & STh/L=    0.595         53 Report
                4  0  0,  10  0  0,  12  0  0,   8  7  0,  12  7  0,   8  8  0,
               10  8  0,   4  9  0,   8  9  0,  10  9  0,   4 10  0,   6 10  0,
                8 10  0,  10 10  0,   6 11  0,  10 11  0,   7  6  1,   5  8  1,
                7  6  2,   7  6  3,   1 16  3,  13  0  4,   6 15  4,   4 16  4,
               13  2  6,  13  2  7,  13  2  8,   0  4  9,   0  4 10,  12  3 11,
                   (   23 More Missing: see the .ckf listing file)
PLAT971_ALERT_2_C Check Calcd Resid. Dens.  1.40Ang From F00Q           2.49 eA-3
```

Author Response: This Alert is due to presence of residual density in the presence of heavy metal atom (Ag).

```
PLAT971_ALERT_2_C Check Calcd Resid. Dens.  1.05Ang From Ag03           2.43 eA-3
```

Author Response: This Alert is due to presence of residual density in the presence of heavy metal atom (Ag).

```
PLAT971_ALERT_2_C Check Calcd Resid. Dens.  1.15Ang From Ag01           2.41 eA-3
```

Author Response: This Alert is due to presence of residual density in the presence of heavy metal atom (Ag).

```
PLAT971_ALERT_2_C Check Calcd Resid. Dens.  0.20Ang From Ag01           2.39 eA-3
```

Author Response: This Alert is due to presence of residual density in the presence of heavy metal atom (Ag).

```
PLAT971_ALERT_2_C Check Calcd Resid. Dens.  1.91Ang From Ag03           2.33 eA-3
```

Author Response: This Alert is due to presence of residual density in the presence of heavy metal atom (Ag).

```
PLAT971_ALERT_2_C Check Calcd Resid. Dens.  1.00Ang From Ag02           2.33 eA-3
```

Author Response: This Alert is due to presence of residual density in the presence of heavy metal atom (Ag).

```
PLAT971_ALERT_2_C Check Calcd Resid. Dens.  0.23Ang From Ag02           2.32 eA-3
```

Author Response: This Alert is due to presence of residual density in the presence of heavy metal atom (Ag).

```
PLAT971_ALERT_2_C Check Calcd Resid. Dens.  0.56Ang From O00E           2.21 eA-3
```

Author Response: This Alert is due to presence of residual density in the presence of heavy metal atom (Ag).

```
PLAT971_ALERT_2_C Check Calcd Resid. Dens.  0.91Ang From Ag01           2.20 eA-3
```

Author Response: This Alert is due to presence of residual density in the presence of heavy metal atom (Ag).

```
PLAT971_ALERT_2_C Check Calcd Resid. Dens.  0.87Ang From Ag02           2.17 eA-3
```

Author Response: This Alert is due to presence of residual density in the presence of heavy metal atom (Ag).

```
PLAT971_ALERT_2_C Check Calcd Resid. Dens.  1.81Ang From N00L           2.15 eA-3
```

Author Response: This Alert is due to presence of residual density in the presence of heavy metal atom (Ag).

```
PLAT971_ALERT_2_C Check Calcd Resid. Dens.  1.42Ang From N00R           2.00 eA-3
```

Author Response: This Alert is due to presence of residual density in the presence of heavy metal atom (Ag).

```
PLAT971_ALERT_2_C Check Calcd Resid. Dens.  0.97Ang From F00Q           1.99 eA-3
```

Author Response: This Alert is due to presence of residual density in the presence of heavy metal atom (Ag).

```
PLAT971_ALERT_2_C Check Calcd Resid. Dens.  1.07Ang From Ag01           1.97 eA-3
```

Author Response: This Alert is due to presence of residual density in the presence of heavy metal atom (Ag).

```
PLAT971_ALERT_2_C Check Calcd Resid. Dens.  1.15Ang From Ag02           1.94 eA-3
```

Author Response: This Alert is due to presence of residual density in the presence of heavy metal atom (Ag).

```
PLAT972_ALERT_2_C Check Calcd Resid. Dens.  1.00Ang From Ag01          -2.47 eA-3
```

Author Response: This Alert is due to presence of residual density in the presence of heavy metal atom (Ag) and disordered structure.

```
PLAT972_ALERT_2_C Check Calcd Resid. Dens.  1.12Ang From Ag01          -2.40 eA-3
```

Author Response: This Alert is due to presence of residual density in the presence of heavy metal atom (Ag) and disordered structure.

```
PLAT972_ALERT_2_C Check Calcd Resid. Dens.  1.12Ang From Ag01          -2.40 eA-3
```

Author Response: This Alert is due to presence of residual density in the presence of heavy metal atom (Ag) and disordered structure.

```
PLAT977_ALERT_2_C Check Negative Difference Density on H01E      .      -0.60 eA-3
```

Alert level G

```
PLAT003_ALERT_2_G Number of Uiso or U(i,j)  Restrained non-H-Atoms          7 Report
PLAT072_ALERT_2_G SHELXL First  Parameter in WGHT  Unusually Large       0.20 Report
PLAT186_ALERT_4_G The CIF-Embedded .res File Contains ISOR Records          4 Report
PLAT242_ALERT_2_G Low    'MainMol' Ueq as Compared to Neighbors of       C01B Check
PLAT242_ALERT_2_G Low    'MainMol' Ueq as Compared to Neighbors of       C01C Check
PLAT242_ALERT_2_G Low    'MainMol' Ueq as Compared to Neighbors of       C01F Check
PLAT242_ALERT_2_G Low    'MainMol' Ueq as Compared to Neighbors of       C01G Check
PLAT242_ALERT_2_G Low    'MainMol' Ueq as Compared to Neighbors of       C01I Check
PLAT242_ALERT_2_G Low    'MainMol' Ueq as Compared to Neighbors of       C01J Check
PLAT720_ALERT_4_G Number of Unusual/Non-Standard Labels ..........         73 Note
              Ag01    Ag02    Ag03    F004    F005    F006    F007    F008
              F009    F00A    F00B    F00C    F00D    O00E    O00F    F00G
              F00H    F00I    N00J    N00K    N00L    N00M    F00N    N00O
```

```
              F00P    F00Q    N00R    F00S    F00T    C00U    H00U    C00V
              H00A    H00B    C00W    C00X    H00X    C00Y    C00Z    H00Z
              C010    H010    C011    H01A    H01B    C012    C013    C014
              C015    C016    H01C    H01D    C017    C018    C019    C01A
              C01B    C01C    C01D    H01E    H01F    C01E    H01G    C01F
              C01G    C01H    H01H    C01I    C01J    C01K    H01I    H01J
              H01K
PLAT860_ALERT_3_G Number of Least-Squares Restraints ............         42 Note
PLAT883_ALERT_1_G Absent Datum for _atom_sites_solution_primary ..     Please Do !
PLAT909_ALERT_3_G Percentage of I>2sig(I) Data at Theta(Max) Still        83% Note
PLAT910_ALERT_3_G Missing FCF Reflection(s) Below Theta(Min)[Deg]=       4.49 Note
                0  0  2,   0  0  4,
PLAT913_ALERT_3_G Missing # of Very Strong Reflections in FCF ....          1 Note
                4  0  0,
PLAT933_ALERT_2_G Number of HKL-OMIT Records in Embedded .res File          5 Note
                0  0  4,   0  0 18,   1 16  3,   4 16  4,   6 15  4,
PLAT941_ALERT_3_G Average HKL Measurement Multiplicity ..........        2.9 Low
PLAT969_ALERT_5_G The 'Henn et al.' R-Factor-gap value ..........     12.573 Note
              Predicted wR2: Based on SigI**2  2.79 or SHELX Weight 21.32
PLAT978_ALERT_2_G Number C-C Bonds with Positive Residual Density.          0 Info
```

```
  7 ALERT level A = Most likely a serious problem - resolve or explain
 26 ALERT level B = A potentially serious problem, consider carefully
 27 ALERT level C = Check. Ensure it is not caused by an omission or oversight
 19 ALERT level G = General information/check it is not something unexpected

  3 ALERT type 1 CIF construction/syntax error, inconsistent or missing data
 63 ALERT type 2 Indicator that the structure model may be wrong or deficient
  9 ALERT type 3 Indicator that the structure quality may be low
  3 ALERT type 4 Improvement, methodology, query or suggestion
  1 ALERT type 5 Informative message, check
```

It is advisable to attempt to resolve as many as possible of the alerts in all categories. Often the minor alerts point to easily fixed oversights, errors and omissions in your CIF or refinement strategy, so attention to these fine details can be worthwhile. It is up to the individual to critically assess their own results and, if necessary, seek expert advice.

Validation response form

Please find below a validation response form (VRF) that can be filled in and pasted into your CIF.

```
# start Validation Reply Form
_vrf_DIFMX02_lcz033
;
PROBLEM: The maximum difference density is > 0.1*ZMAX*0.75
RESPONSE: ...
;
_vrf_PLAT042_lcz033
;
PROBLEM: Calc. and Reported MoietyFormula Strings  Differ     Please Check
RESPONSE: ...
;
_vrf_PLAT082_lcz033
```

```
;
PROBLEM: High R1 Value ..................................       0.12 Report
RESPONSE: ...
;
_vrf_PLAT084_lcz033
;
PROBLEM: High wR2 Value (i.e. > 0.25) ...................       0.35 Report
RESPONSE: ...
;
_vrf_PLAT234_lcz033
;
PROBLEM: Large Hirshfeld Difference F00P     --C01C     .       0.16 Ang.
RESPONSE: ...
;
_vrf_PLAT342_lcz033
;
PROBLEM: Low Bond Precision on  C-C Bonds ...............    0.01725 Ang.
RESPONSE: ...
;
_vrf_PLAT906_lcz033
;
PROBLEM: Large K Value in the Analysis of Variance ......      4.403 Check
RESPONSE: ...
;
_vrf_PLAT911_lcz033
;
PROBLEM: Missing FCF Refl Between Thmin & STh/L=    0.595         53 Report
RESPONSE: ...
;
_vrf_PLAT977_lcz033
;
PROBLEM: Check Negative Difference Density on H01E      .      -0.60 eA-3
RESPONSE: ...
;
# end Validation Reply Form
```

PLATON version of 17/09/2025; check.def file version of 17/09/2025

---

duplicate check

No duplication found

Datablock lcz033 - ellipsoid plot

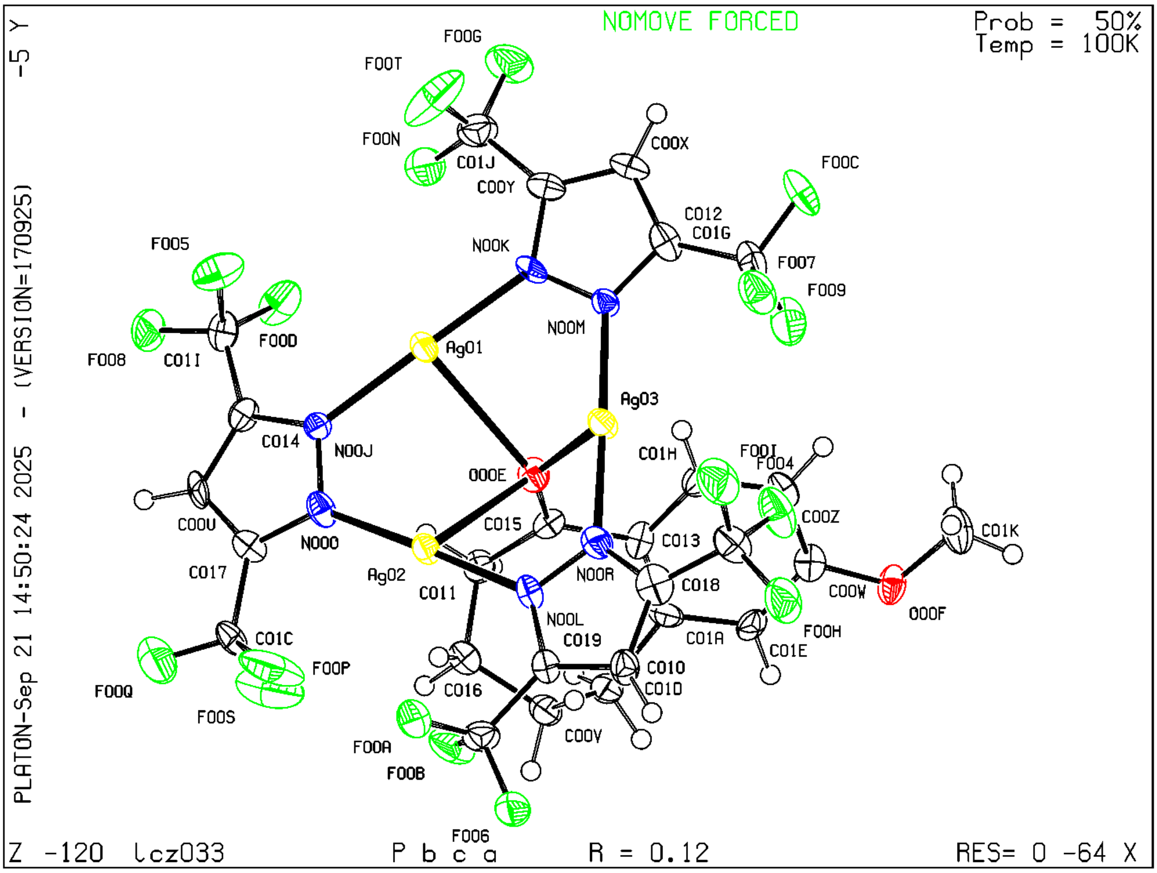

Supplement: Supplementary file 2 — Supporting File 2: anie72462‐sup‐0002‐DataFile.zip. [file ANIE-65-e1218503-s002.zip › supplementary_materials_crystallographic_data submit.zip!/cif_and_checkcif(1-114)/Ag3Pz3_33/Ag3Pz3_33_checkcif.pdf]
